# HD-ZIP Transcription Factors and Brassinosteroid Signaling Play a Role in Capitulum Patterning in Chrysanthemum

**DOI:** 10.3390/ijms24087655

**Published:** 2023-04-21

**Authors:** Annemarie Castricum, Erin H. Bakker, Nick C. M. H. de Vetten, Mieke Weemen, Gerco C. Angenent, Richard G. H. Immink, Marian Bemer

**Affiliations:** 1 Bioscience, Wageningen University & Research, 6700 AA Wageningen, The Netherlands; 2 Laboratory of Molecular Biology, Wageningen University & Research, 6700 AA Wageningen, The Netherlands; 3 Dekker Chrysanten, 1711 RP Hensbroek, The Netherlands

**Keywords:** Chrysanthemum, capitulum, disc floret, ray floret, HD-ZIP IV, brassinosteroids

## Abstract

Chrysanthemum is a genus in the Asteraceae family containing numerous cut flower varieties with high ornamental value. It owes its beauty to the composite flower head, which resembles a compact inflorescence. This structure is also known as a capitulum, in which many ray and disc florets are densely packed. The ray florets are localized at the rim, are male sterile, and have large colorful petals. The centrally localized disc florets develop only a small petal tube but produce fertile stamens and a functional pistil. Nowadays, varieties with more ray florets are bred because of their high ornamental value, but, unfortunately, this is at the expense of their seed setting. In this study, we confirmed that the disc:ray floret ratio is highly correlated to seed set efficiency, and therefore, we further investigated the mechanisms that underlie the regulation of the disc:ray floret ratio. To this end, a comprehensive transcriptomics analysis was performed in two acquired mutants with a higher disc:ray floret ratio. Among the differentially regulated genes, various potential brassinosteroid (BR) signaling genes and HD-ZIP class IV homeodomain transcription factors stood out. Detailed follow-up functional studies confirmed that reduced BR levels and downregulation of HD-ZIP IV gene *Chrysanthemum morifolium PROTODERMAL FACTOR 2* (*CmPDF2*) result in an increased disc:ray floret ratio, thereby providing ways to improve seed set in decorative chrysanthemum varieties in the future.

## 1. Introduction

The chrysanthemum, the second most important cut flower in the world, appeals to many people because it has a diverse range of flower shapes and colors, owing to its composite flowers that are characteristic of the Asteraceae family. A composite flower head resembles a typical flower but is a compressed inflorescence structure (capitulum) where many different individual flowers, known as florets, are packed together and surrounded by involucral bracts [1,2]. Depending on the species, the Asteraceae capitulum may contain only disc florets, only ray florets, or a combination of both, with centrally the disc and marginally the ray florets. Disc florets are hermaphroditic actinomorphic florets that first produce pollen and later develop a functional pistil. Ray florets are zygomorphic, with showy petals fused to a ligule, and generally do not develop stamen. Some chrysanthemum cultivars contain florets intermediate between ray and disc florets, which are called trans florets. Originally, chrysanthemum varieties had flowers with many disc florets at the center of the capitulum and only a single row of ray florets at the rim, presumably to help attract insects for pollination [3]. After many years of successful breeding, chrysanthemum varieties grown today vary substantially in the ray:disc floret ratio. Some varieties have ray florets only at the rim of the capitulum (daisy-type), while in other varieties, the ray florets cover most of the capitulum (decorative-type). Generally, the total number of florets on the capitulum is not changed in these varieties but only in floret identity and, consequently, the disc:ray floret ratio. Varieties with more ray florets are considered to have higher ornamental value, but a serious drawback is that these varieties usually display severe fertility and seed set issues [4]. Therefore, more knowledge on the mechanisms determining floret identity or the regulation of the disc:ray floret ratio is highly desired. 

Several studies have aimed at elucidating the mechanism responsible for the determination of floret identity in Asteraceae. An important role in regulating ray floret identity appears to be fulfilled by *CYCLOIDEA(CYC)-*like TCP transcription factors [5]. In Asteraceae species, the *CYC* genes have undergone several duplication events leading to three clades, *CYC1*, *CYC2,* and *CYC3*, which can function in complexes composed of dimers of CYC proteins from the same or different CYC clades [6]. In several species, including chrysanthemum, sunflower, gerbera, and *Senecio vulgaris*, overexpression of a *CYC2-*like gene increased ligule length in ray florets, and in both gerbera and *Senecio*, it disrupted stamen development in disc florets [3,7,8,9,10,11]. In gerbera, *GhCYC2* is highly expressed in the marginal ray florets but not expressed in the central disc flowers, and its downregulation reduces ligule length in the intermediate trans flowers [7]. Particularly in sunflowers, the role of *CYC2* has been well-studied, and it appeared to be a major determinant of mature floret phenotype. Overexpression of *HaCYC2c*, induced by the insertion of a transposable element in the promoter, results in capitula that contain only ray florets [8]. On the other hand, the malfunction of *HaCYC2c* in the sunflower *tub* mutants results in functional pistil and stamen development in the normally sterile ray florets [8]. Similarly, expression of the *CYC2*-like genes *RAY1, RAY2,* and *RAY3* is restricted to the ray floret region in *Senecio* and regulates the development of ray florets [3,12]. This activity includes the zygomorphic outgrowth of the ray floret petals, which is fulfilled in a complex interaction with the MYB-domain transcription factor genes *RADIALIS* (*RAD*) and *DIVARICATA* (*DIV*) [12]. In chrysanthemums, evidence for the roles of *CYC2-*like genes in petal outgrowth has been presented. Overexpression of *CYC2c* has a positive effect on ligule length in both disc and ray florets [13], whereas overexpression of *CYC2d* has a negative effect on ray-floret length [10]. Furthermore, the knock-down of *ClCYC2g* in wild-type *Chrysanthemum lavandulifolium* led to the disruption of ligule outgrowth in ray florets [14]. In severely down-regulated lines, this appeared to be concomitant with the reappearance of stamens, which sometimes developed functional pollen [14]. Thus, also in chrysanthemums, *CYC2-*like genes are involved in ray floret development, although the exact contribution of the various gene copies is not yet clear.

To obtain a more in-depth understanding of the differential development of disc and ray florets in chrysanthemums, various comparative transcriptomics studies, and targeted expression analyses focusing on previously described flower development genes have been performed [15,16,17,18,19,20,21,22]. Putative chrysanthemum ABCE-class MADS-box genes *APETALA1* (*AP1*, A-function) and *AGAMOUS* (*AG*, C-function) were found to be higher expressed in disc florets, while *APETALA3* (*AP3*, B-function) had increased expression in ray florets [19]. Note that in most of these studies, the transcriptomes of late developmental stages of ray and disc florets have been compared when floret identity is already established. Consequently, the identified differentially expressed genes reflect the phenotypic differences between ray and disc florets rather than being related to the regulation of their identity. This holds even for the *CYC2-*like genes, which seem to be involved in the establishment of ray floret identity shortly after pre-setting of the floret identity [23]. These genes, and other identity-establishing genes, are most likely acting directly downstream of patterning genes or hormones that establish the region in which the different floret types will develop. However, how the patterning of this region is orchestrated is still largely unresolved. 

To investigate the mechanisms and factors underlying the regulation of capitulum patterning in chrysanthemums, we identified two mutants in different genetic backgrounds with a substantially higher disc:ray floret ratio than the original variety. Exploring this material, we collected a time series of RNA samples from early developmental capitulum stages up to the moment that floret types started to deviate phenotypically. A subsequent differential transcriptomics analysis resulted in a list of differentially expressed genes (DEGs), including various brassinosteroid (BR) signaling genes and HD-ZIP class IV homeodomain transcription factors. Detailed functional analyses provided evidence that reduced BR signaling and reduced levels of the HD-ZIP IV gene *Chrysanthemum morifolium PROTODERMAL FACTOR 2 (CmPDF2)* both resulted in the expansion of the region with disc florets. In the future, this knowledge could provide ways to control and steer these important ornamental traits and facilitate the breeding and production of new decorative-type chrysanthemum varieties.

## 2. Results

### 2.1. Chrysanthemum Types with Less Disc Florets Produce Fewer Seeds

**Figure 1:**
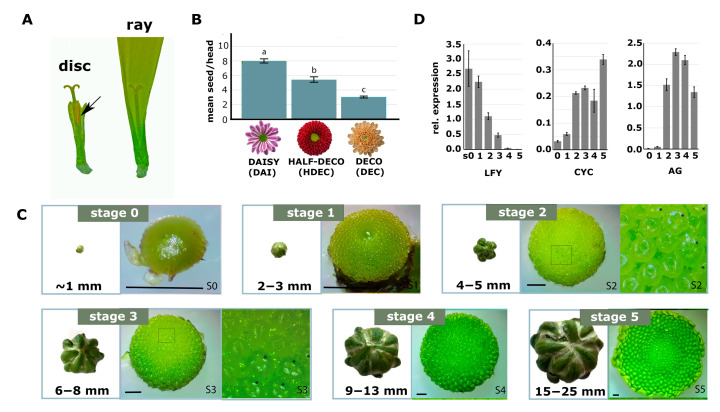
Chrysanthemum capitulum development and the correlation between seed set efficiency and the relative number of disc florets in the capitulum. (**A**) Morphology differences between a full-grown mature disc floret (left) and ray floret (right). The corolla tube of the disc floret was partially removed to display the anthers (arrow); the ray floret ligule was cut at the top. (**B**) Representative examples of a Daisy-type (DAI), Half-Decorative-type (HDEC), and a Decorative-type (DEC) capitulum, going from mainly disc florets to mainly ray florets, respectively. Per type, the average seed set on a single capitulum is shown upon hand pollination with the same amount of pollen. (**C**) Six subsequent developmental stages (S0–S5) of a chrysanthemum capitulum. For each stage, the closed capitulum bud is shown on the left. Next to it on the right, dissected capitulum buds are shown in which the initial stages of floret development are visible. For S2 and S3, part of the capitulum (boxed region) is enlarged. In this zoom-in representation, the radially symmetrical disc florets (marked with an asterisk) can be distinguished from zygomorphically symmetrical ray florets. (**D**) Expression of *LFY*, *AG*, and *CYC2c* in the six capitulum developmental stages as determined by qRT-PCR. Significance in (**B**) determined by ANOVA, *p* < 0.05. Error bars represent standard error. Number of measurements: DAI, 7486; HDE, 3086; DEC, 17,987. Size bar in (**C**) = 1 mm.

Chrysanthemum has two floret types: disc and ray florets, of which the latter do not develop anthers but contain a very long petal called a ligule (Figure 1A). Both disc and ray florets develop female reproductive organs, including pistils, comprising of stigma, style, and ovary. Several Asteraceae species exhibit reduced fertility or complete sterility of ray florets [1], and we hypothesized that chrysanthemum varieties with a relatively high number of disc florets in comparison to the number of ray florets have better seed sets. To get insight into the relationship between floret type and seed set, data from an extensive collection of crosses between varieties with different percentages of disc florets were analyzed. The hand-pollinations for these crosses were performed with similar amounts of pollen. To facilitate the analysis, the data were grouped into three categories based on the composition of florets in the capitulum of the mother plant: (I) Daisy types, containing mostly disc florets and a limited number of ray florets at the rim of the capitulum, (II) Half-Decorative types, having more or less similar numbers of disc and ray florets, and (III) Decorative types, containing mostly ray florets and only a limited number of disc florets in the center of the capitulum (Figure 1B). A positive correlation between the relative number of disc florets (capitulum type) and seed set was found, confirming that the disc:ray floret ratio affects seed set efficiency in chrysanthemums (Figure 1B). 

To unravel the mechanism responsible for floret identity determination and, consequently, disc:ray floret ratio in the mature capitulum, we first investigated by microscopic observations at which moment floret identity is established (Figure 1C). Capitulum buds of different developmental stages were dissected under the microscope and imaged. At stage 0 (S0), the capitulum represents an initial inflorescence meristem (IM) with a smooth appearance. At S1, part of the IM was replaced by developing floret primordia, which developed from outside to inside. The IM was completely filled by floret primordia at S2. This was also the first stage at which differences could be observed between ray and disc floret primordia (Figure 1C). This difference became more apparent in S3. At S4, all florets had differentiated, and ray and disc florets were easily distinguishable. At S5, ligules even started to color (Figure 1C).

To obtain more detailed insight into the development of the capitulum and florets and to support and validate our division in six developmental stages (S0–S5), the expression of three key marker genes was investigated by qRT-PCR. *LEAFY (LFY)* is an essential gene involved in capitulum development, which in chrysanthemums was shown to be lowly expressed in vegetative meristems and strongly induced upon IM initiation [17]. Research in gerbera showed that *LFY* is the master regulator of IM identity and is primarily expressed in the undifferentiated smooth capitulum dome and involucral bracts. Subsequently, it was also detected in the emerging floret primordia [24]. In line with these observations, we found the highest expression of *LFY* at S0, which decreased upon floret primordia initiation (S1) and even further decreased during subsequent floret differentiation (S2–S5; Figure 1D). Secondly, the expression of *CYCLOIDEA (CYC),* a TCP transcription factor gene responsible for the outgrowth of floret tissues, was investigated. Studies in chrysanthemums have shown that *CYC2c* expression is especially high when the ligules start to expand [13,25]. We found that *CYC2c* expression strongly increases at S2 and rises further at S5, the moment that ligule outgrowth and coloring become apparent (Figure 1D). Finally, the expression of the C-class MADS-box gene *AGAMOUS (AG)* was investigated. *AG* determines stamen and carpel identity in floret primordia and has previously been found to be expressed when the reproductive organs are initiated [26]. As expected, we found its expression being initiated at S2 and maintained till S5 (Figure 1D). In conclusion, our observations revealed that disc and ray florets started to develop visually differently from S2 onwards and, therefore, that the initial molecular establishment of floret identity should take place in the timespan before S2.

### 2.2. Identification and Characterization of a Mutant with Altered Disc:Ray Floret Ratio

**Figure 2:**
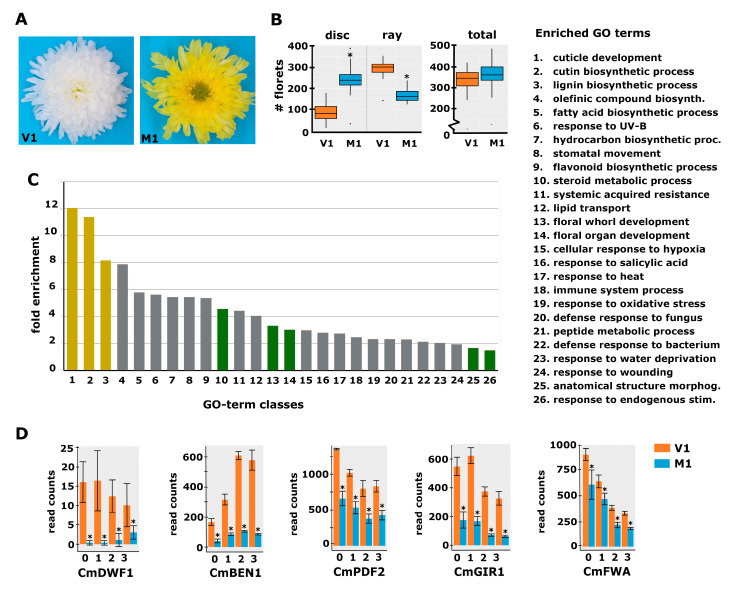
Phenotypic characterization of a spontaneous chrysanthemum mutant with decreased disc:ray floret ratio. (**A**) Top view of a mature capitulum from variety 1 (V1; left) and its spontaneous mutant (M1; right) that showed a strong increase in the number of disc florets and yellow instead of white ligules. (**B**) Boxplot showing the number of disc and ray florets and the total number of florets in V1 and M1 capitula (*n* = 7). (**C**) GO-term enrichment analysis with the DEGs of M1 vs. V1 at stages 0 and 1. The legend for the category numbers is displayed on the right. The *y*-axis displays the fold enrichment of the number of genes in the category compared to the Arabidopsis genome; the *x*-axis displays the different significantly enriched categories (FDR < 0.05). The categories discussed in the text are displayed in yellow and green. (**D**) Expression plots for five selected genes from the RNA-seq experiment (RPKM read count values are displayed). The transcript identifiers are as follows: *CmDWF1*, DN96938; *CmBEN1*, DN45086; *CmPDF2*, DN65126; *CmGIR1*, DN43323; and *CmFWA*, DN60377. The numbers at the *x*-axis indicate the stages. Error bars indicate the SE of the three biological replicates; asterisks indicate significance at Padj < 0.01.

To identify genes that regulate the early establishment of floret identity and thereby determine the disc:ray floret ratio, we searched for mutants in decorative varieties that displayed an increased disc:ray floret ratio compared to the original variety. We identified a spontaneous mutant that contained significantly more disc florets than its corresponding genetic background variety (V1). V1 has a white decorative flower with mostly showy ray florets and only a limited number of disc florets in the middle of the capitulum (Figure 2A). Its spontaneous mutant (M1) is yellow and has considerably fewer ray florets and more disc florets (Figure 2A). To quantify the effect, the total number of florets and the number of ray and disc florets were counted in the capitula of seven plants from V1 and M1. This analysis revealed that the total floret number is unchanged in the mutant, while the disc:ray floret ratio increased from 0.3 in V1 to 1.47 in M1 (Figure 2B). 

Subsequently, we decided to perform a transcriptome analysis to identify genes differentially expressed in M1 vs. V1 at different early capitulum stages (S0–S3). To obtain a useful reference chrysanthemum transcriptome, a de novo transcriptome assembly was generated using Trinity software based on all RNA-seq reads of the V1 samples. This resulted in an assembly consisting of 746,130 isoforms, representing 251,805 putative genes. This high number of genes is not surprising, given the hexaploidy and highly heterozygous nature of the chrysanthemum genome in conjunction with using short sequence reads. However, it complicates the assessment of whether two related transcripts represent different genes or different alleles of the same gene. BLAST against the Arabidopsis and sunflower references was used for annotation, which was successful for 62% of the assembled sequences. This annotation could be further improved through individual BLAST searches of various unannotated transcripts against all sequences in the NCBI database. The remaining unidentified sequences were short and most likely incomplete or incorrectly assembled sequences. To validate if low abundance transcripts were represented in this chrysanthemum reference, we investigated whether sequences representing the potential orthologs of the genes *WUSCHEL (WUS), BRANCHED1 (BRC1/TCP18), CLAVATA3 (CLV3), LFY, NUBBIN (NUB), SQUAMOSA PROMOTER BINDING PROTEIN-LIKE8 (SPL8),* and *JAGGED (JAG)* could be identified. These genes were selected because of their defined and very local expression. From this selection, all genes except *NUB* could be identified, giving us confidence in the quality and completeness of the generated chrysanthemum reference.

### 2.3. Differential Expression Analysis Reveals Genes Possibly Involved in the Regulation of the Disc:Ray Floret Ratio

To identify candidate genes for floret identity determination and establishment of the disc:ray floret ratio, samples from a developmental time series of S0 to S3 capitula (see Figure 1C) of V1 and M1 plants were harvested, followed by RNA-seq analysis. The obtained reads were mapped against the generated reference transcriptome and quantified using RSEM and Bowtie2. The average mapping efficiency was 70.2%, resulting in, on average, 29.3 million mapped read pairs per sample. Because the phenotypic difference between V1 and M1 was of a quantitative nature, we reasoned that the expression differences of genes involved in the regulation of the disc:ray floret ratio may only be mild. Therefore, we called differentially expressed genes (DEGs) solely based on the significant difference between the three biological replicates of M1 and V1 (adjusted *p*-value < 0.01), regardless of the fold change. We identified 887, 1032, 1323, and 981 DEGs in the stages S0, S1, S2, and S3, respectively, of which the vast majority were downregulated in the mutant (Table S1). The *CAROTENOID CLEAVAGE DIOXYGENASE 4a* (*CCD4a*) gene, known to be responsible for the conversion of chrysanthemum flower color from white to yellow [27], also appeared among the DEGs, in accordance with the color change of the mutant ligules. To get more insight into the genes and pathways that may underlie the regulation of disc:ray floret ratio, we performed a gene ontology enrichment analysis (GO analysis) using the differentially expressed genes (DEGs) from S0 and S1 only. Only the genes having an annotation based on Arabidopsis were included in this analysis. In total, 676 annotated transcripts were differential between V1 and M1 at S0 and/or S1. Several of the enriched transcripts had the same annotation based on Arabidopsis and may thus represent highly homologous chrysanthemum genes or different alleles of the same gene. After filtering these out, 531 unique ‘gene identifiers’ remained for the GO enrichment analysis, which was performed using PANTHER. Focusing on the ‘biological processes’ dataset, 26 categories were found to be significantly overrepresented among the DEGs (Figure 2C). The three most prominent categories were associated with cuticle, cutin, and lignin synthesis, three related pathways [28]. This shows, together with the differential expression of the *CCD4a* gene, that multiple phenotypic alterations have occurred in the mutant in addition to the change in the disc:ray floret ratio.

With this in mind, we focused on categories that can be linked to the regulation of tissue identity and development based on studies in a wide range of plant species. These included the GO-terms ‘Response to endogenous stimuli, Anatomical structure morphogenesis, and Floral organ- and Floral whorl development’, all of which are relevant in relation to the establishment of specific floret identity. In addition, the group ‘Steroid metabolic process’ was enriched, comprising various genes for brassinosteroid (BR) biosynthesis (Table S2a). Since BRs have been associated with organ growth and development, and plant reproduction [29], we considered this group interesting as well. The most prominent gene in the ‘steroid metabolic process’ group was *DWARF1 (DWF1)*, encoding for an enzyme regulating the conversion of the precursor 24-methylenecholesterol to campesterol early in the BR biosynthesis pathway [30]. The chrysanthemum *DWF1* homolog *CmDWF1* was downregulated in the S0 and S1 capitulum of M1 with log2 fold changes of 5.4 and 5.8, respectively (Figure 2D). In addition to *DWF1*, *BRI1-5 ENHANCED 1* (*BEN1*), a dihydroflavonol 4-reductase with a role in BR accumulation [31,32], belongs to the ‘steroid metabolic process’ group. The chrysanthemum homolog of this gene, *CmBEN1,* was downregulated at both S0 (log2fold = 2.2) and S1 (log2fold = 2.1) (Figure 2D). Downregulation of these BR biosynthesis genes suggests that the production of BRs is disturbed in the mutant, a hypothesis that was further strengthened by the differential expression of several genes from the significantly enriched ‘Response to endogenous stimuli’ group (Table S2b). This group includes the BR receptor kinase *HERCULES 1 (HERK1)*, of which two homologous chrysanthemum transcripts were strongly downregulated at both early capitulum developmental stages (log2fold changes 3.3–5.5). A homolog of *FERONIA* (*FER*), another receptor-like kinase responsive to BR [33], turned out to be strongly downregulated (log2fold S0 = 6.4 and S1 = 6.2). *BRI1-ASSOCIATED RECEPTOR KINASE* (*BAK1*) was also present in this group of genes, but instead of being downregulated, the homologous chrysanthemum transcripts were mildly upregulated in S0 (log2fold = −1.5), potentially compensating for the strong downregulation of *CmHERK1* and *CmFER*. A few cytokinin- and auxin response regulators were also present in the ‘Response to endogenous stimuli’ group, but their up- or downregulation was mild compared to that of the BR regulators. It is important to note that for some genes, such as *CmDWF1* and *CmHERK1*, different chrysanthemum transcripts were assembled, which may either represent different genes or different alleles of the same gene. While these sometimes displayed a similar differential expression, such as in the case of the *CmHERK1* transcripts (DN49627 and DN68032), they had different patterns in other cases, such as for the *CmDWF1* transcripts DN47421, DN45082, and DN96938, where only the last one showed a strong downregulation in M1. Altogether, the fact that both BR biosynthesis and BR receptor genes are strongly differentially regulated in M1 suggests a role for BR in the establishment of floret identity early in capitulum development. To further search for candidates that may underlie the specification of floret identity, we took a closer look at the genes in the groups ‘Floral organ development, Floral whorl development, and Anatomical structure morphogenesis’. This investigation resulted in a list of 13 potentially interesting transcription factors, including four homeodomain zipper proteins (HD-ZIP) [34] and an interactor of HD-ZIP IV proteins [35,36], which are all 1.5 to 2.5 times downregulated in the mutant (Figure 2D; Table S3). Because it is difficult to assess solely based on annotation which of these genes are proper candidates for the regulation of disc:ray floret ratio, we decided to involve another variety and corresponding mutant that displayed an altered disc:ray floret ratio.

### 2.4. Differential Gene Expression in a Second Genetic Background with Altered Disc:Ray Floret Ratio

**Figure 3:**
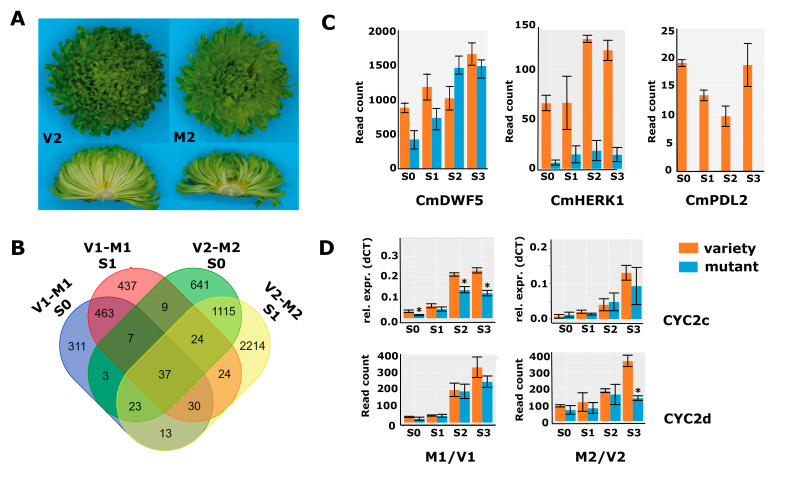
DEG analysis in a second genetic background. (**A**) Pictures of variety 2 (left panels) and mutant 2 (right panels) of a complete inflorescence (top) and transversely sectioned inflorescence (bottom). (**B**) Overlap of DEGs between V1/M1 and V2/M2 in stages 0 and 1. (**C**) Read count data from the second RNA-seq experiment (V2/M2) for *CmDWF5* (DN57668), *CmHERK1* (DN68032), and *CmPDL2* (DN38497). (**D**) qPCR data for *CYC2c* and read count data for *CYC2d* from both RNA-seq experiments (V1/M1 left; M2/V2 right). Error bars indicate SE; asterisks indicate significance (qPCR: *p* < 0.05; RNA-seq: Padj < 0.01).

A second mutant of interest was obtained in a mutant screen after X-ray treatment of cuttings of variety 2 (V2) with 17.5 Gy. V2 has green flowers and a decorative flower type with many ray florets (Figure 3A). The percentage of disc florets in V2 was 6.4%, which increased to 33.4% in M2, representing a 5.2-fold increase. The average total floret number per capitulum showed a slight increase from 315 (V2) to 357 (M2), in contrast to V1/M1, where the total number of florets had not changed. Capitulum material from a developmental time series (S0–S3; Figure 1C) was harvested and further processed for RNA-seq. Obtained reads were mapped against the generated V1 chrysanthemum transcriptome and quantified in the same way as done for V1/M1. The number of differentially expressed genes for V2/M2 was much higher than for V1/M1, reflecting the different nature of the mutants, with M1 being a spontaneous mutant and M2 descending from X-ray treatment, which is known to induce many smaller and larger deletions [37]. The differential gene expression analysis revealed 1859, 3477, 7162, and 7428 DEGs in the stages S0, S1, S2, and S3, respectively (Table S4). 

The overlap in DEGs between V1/M1 and V2/M2 in stages 0 and 1 was low (Figure 3B), showing that there are indeed many non-related processes disturbed in both mutants. However, as the disc:ray floret ratio phenotypes were similar, we expected some overlap in differential gene expression that could aid in further delineating the number of candidate genes for functional follow-up studies. To get more insight into putative overlapping modified pathways, we also performed the GO enrichment analysis for the V2/M2 DEGs from stages 0 and 1 based on the Arabidopsis annotations. The list with significantly enriched GO terms was long, with 50 enriched main terms, and showed only a modest overlap with the 26 categories enriched for V1/M1 (Table S5). We, therefore, searched specifically in the enriched categories ‘Regulation of hormone levels’ and ‘pattern specification process/anatomical structure development’. The first group contained a mix of genes involved in cytokinin, auxin, and brassinosteroid signaling, which, together with the significant enrichment of the ‘gibberellin-mediated signaling pathway’, suggests that hormone homeostasis is more generally affected in M2. Focusing on the BR-related genes, we discovered that also in M2, a brassinosteroid biosynthesis gene (homolog of DWF5) and homologs of the BR receptor kinase HERK1 were significantly downregulated at early stages of capitulum development (Figure 3C). Thus, although these concerned different transcripts/genes, BR-signaling is likely compromised during early capitulum development in M2 as well. Interestingly, also four HD-ZIP genes were lower expressed at stages 0 and 1 in M2, including homologs of *PROTODERMAL FACTOR 2* (*PDF2), GLABRA2-INTERACTING REPRESSOR 1 (GIR1),* and *HOMEOBOX ARABIDOPSIS THALIANA 4* (*HAT4)*, which were also identified in M1. Most remarkable, however, was the HD-ZIP IV homolog of *ARABIDOPSIS THALIANA MERISTEM LAYER 1* (*ATML1*), which was strongly downregulated in all four sampled stages (log2fold S0 = 6.3 and S1 = 6.1) (Figure 3C, renamed into *CmPDL2*). We then performed a maximum likelihood analysis to assess whether the identified HD-ZIP sequences all grouped in the clade of Arabidopsis HD-ZIP IV proteins [34]. Interestingly, the transcripts that were most significantly differentially expressed, DN65126 and DN32354 in M1, and DN38497 in M2, grouped together with ATML1 and PDF2 in one clade. The protein sequences derived from DN65125 and DN32354 turned out to be identical, unveiling that both transcripts most likely represent the same gene. We designated DN65135/DN32354 as *CmPDF2*, DN32648 as *CmPDF2*-like *1* (*CmPDL1*), and the weaker expressed DN38497 as *CmPDL2*. In addition, DN60377 and DN62803 encoded the same protein sequence and grouped into a clade together with HDG7/FWA, while D55406 is quite divergent and appears sister to most other HD-ZIP IV proteins. The corresponding genes were therefore named *CmFWA* (DN60377/DN62803) and *CmHDG2* (DN55406), respectively. Finally, the *HAT4* homolog DN61217 was identified as an HD-ZIP II class protein. None of the other genes present in the list of potentially interesting TFs of V1/M1 (Table S3) were found back in the V2/M2 DEG list, pointing to the HD-ZIP genes as most interesting TF candidates to play a role in the regulation of the disc:ray floret ratio. To validate the RNA-seq data, we confirmed several of the interesting genes with qPCR in independent samples in either M1/V1 or M2/V2, which revealed that the observed downregulation of brassinosteroid-related genes and Class IV HD-ZIP proteins was consistent in freshly harvested samples (Figure S2).

### 2.5. Cycloidea Genes Are Only Differentially Expressed at Later Stages

*CYCLOIDEA* (*CYC*) encodes a TCP transcription factor responsible for the outgrowth of tissues and is linked to the differential growth of florets in different Asteraceae species [7,23]. Studies in chrysanthemums have shown that *CYC2c* expression is higher in ray florets because it is particularly important for ligule growth [13,25]. We tested the expression of the *CYC2a-f* variants mentioned in these papers in ray and disc florets of different varieties (Figure S3A). This revealed that *CYC2c* and *CYC2d* were both relatively highly expressed and consistently much higher in ray than in disc florets. In addition, *CYC2c* and *CYC2d* had considerably higher expression from stage 2 onwards (Figure 3D), consistent with a role in the outgrowth of the ray florets rather than the establishment of their identity. In both M1 and M2, the expression of *CYC2c* and *CYC2d* was lower than in the non-mutated variety, reflecting the lower number of ray florets in both mutants. Interestingly, two *RAD*-like genes, known as targets of CYCs in the establishment of asymmetric flowers, followed the same pattern as *CYC2c/2d* (Figure S3B), supporting emerging evidence that the CYC-RAD module is conserved beyond the Lamiaceae [38,39,40]. Taken together, these data show that *CYC* and *RAD* genes are only differentially expressed at later stages of floret primordium development, suggesting that these genes are involved in floret organ development rather than floret identity. This strengthens our hypothesis that BRs and HD-ZIP IV genes may be important at an early stage to regulate the disc:ray floret ratio in chrysanthemums, while the *CYC* genes are important slightly later to ensure differential development of the two floret types.

### 2.6. Functional Analysis for a Selection of Identified Candidate Genes

**Figure 4:**
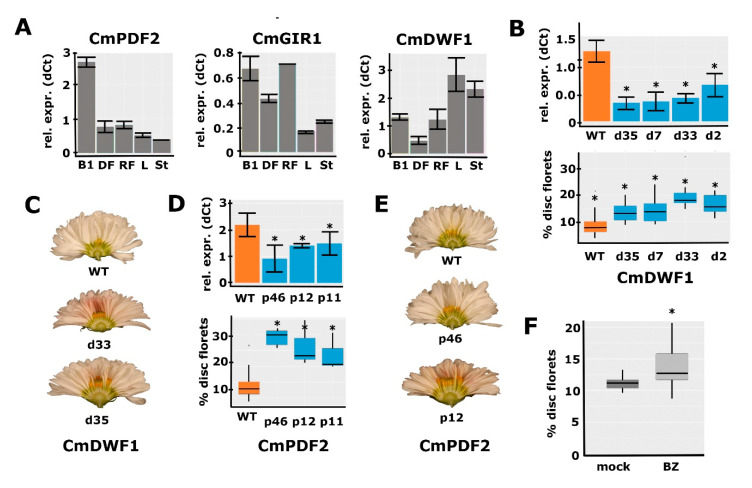
Functional characterization of *CmPDF2*, *CmGIR1*, and *CmDWF1*. (**A**) qPCR analysis of *CmPDF2*, *CmGIR1*, and *CmDWF1* expression in different tissues. B1, bud stage 1; DF, mature disc florets; RF, mature ray florets; L, leaf; St, stem. (**B**) Downregulation of *CmDWF1* transcripts in different RNAi lines (top panel) and the quantification of the corresponding capitulum phenotypes (bottom panel) (**C**) Transverse sections of the capitula of two lines with *CmDWF1* downregulation compared to the WT (top). A higher number of disc florets is visible in the center of the transgenic capitula. (**D**) Downregulation of *CmPDF2* transcripts in different RNAi lines (top panel) and the quantification of the corresponding capitulum phenotypes (bottom panel). (**E**) Transverse sections of the capitula of two lines with *CmPDF2* downregulation compared to the WT (top). A higher number of disc florets is visible in the center of the transgenic capitula. (**F**) Brassinozole treatment of WT inflorescence buds results in a higher number of disc florets. Error bars indicate SE; asterisks indicate significance (qPCR: *p* < 0.05; RNA-seq: Padj < 0.01).

To further study the involvement of brassinosteroid signaling and HD-ZIP IV TFs in the regulation of the disc:ray floret ratio, we selected a few genes from the RNA-seq for downregulation experiments. Because *CmGIR1* was significantly downregulated in both M1 and M2 and annotated as an interactor of HD-ZIP IV TFs [35], we selected it for further functional studies. From the different HD-ZIP IV TFs that were affected in mutants M1/M2, we selected *CmPDF2* (DN65135/DN32354) for downregulation, as it was considerably differentially expressed in M1 and is highly but dynamically expressed during capitulum development, with the highest levels at stages 0 and 1 (Figure 2D). In addition, close homologs of *CmPDF2* were downregulated in M2 (*CmPDL1* (DN32648) and *CmPDL2* (DN38497)). To investigate whether BRs play a role in the establishment of floret identity, we reasoned that impeding BR biosynthesis would be the most effective. We, therefore, selected the *CmDWF1* transcript with the highest abundance in early capitulum development (DN45082) as a target for downregulation. First, we investigated to what extent the expression of the selected genes is specific to reproductive organs. This analysis was performed in *Chrysanthemum morifolium* cultivar ‘1581’, which is transformable [41,42], and expression was compared between leaves, stem, S1 capitula, disc florets, and ray florets. *CmPDF2* and *CmGIR1* expression was higher in reproductive tissues (Figure 4A), supporting a function in floret development. For *CmPDF2,* a particularly high expression was found in the S1 capitulum, representing the stage just prior to the differentiation of the disc and ray floret primordia (Figure 1C). *CmDWF1* was also considerably expressed in reproductive tissues, but its expression was higher in vegetative tissues, indicating that it may be required to produce BRs throughout the plant.

To further investigate the functions of the three selected genes, RNAi downregulation lines were generated in *C. morifolium* cultivar ‘1581’. Based on the differential expression observed in the M1/M2 mutant backgrounds, we hypothesized that transgenic lines with sufficient downregulation of *CmGIR1, CmPDF2,* or *CmDWF1* would mimic the M1/M2 phenotypes and exhibit more disc florets in the capitulum. For each transformation, at least 12 independent transgenic lines were selected, and the downregulation of the targeted gene was tested with qPCR in a sample from a few 0–1 stage buds acquired (Figure S4). In most cases, only a single replicate could be harvested due to the limited number of buds per primary transformant. Although several lines with clear downregulation were obtained for *CmGIR1*, we did not observe consistent differences in the capitulum phenotype associated with the extent of downregulation. This indicates that either the downregulation was not yet sufficient or that *CmGIR1* is not essential for the regulation of the disc:ray floret ratio. For the *CmDWF1* and *CmPDF2* lines, however, downregulation appeared to coincide with an increased disc:ray floret ratio. To produce more reliable measurements, 3–4 individual transformants were selected, of which cuttings were generated so that multiple replicates could be generated for both qPCR analysis and phenotyping (Figure 4B–E). Downregulation of both *CmPDF2* and *CmDWF1* resulted in significantly increased disc:ray floret ratios, with a more pronounced effect of *CmPDF2* downregulation compared to *CmDWF1* downregulation (Figure 4B–E). Besides this effect on the capitulum, no other noticeable phenotypical differences between the transformed lines and controls were observed. For the *DWF1* RNAi lines, no other effects were observed, such as the classical dwarfism phenotype commonly observed in BR-deficient plants. These results provide a strong indication that both a high BR concentration and relatively high *PDF2* expression are required in early bud stages to induce ray floret identity in the emerging primordia.

### 2.7. Confirmation of the Role of BR in Disc:Ray Floret Ratio by Brassinazole Treatments

Since BR is a hormone, we decided to perform further functional investigations by applying exogenous Brassinolide (BL; a synthetic variant of BR), and Brassinazole (BZ; a BR inhibitor), to *C. morifolium* cultivar ‘1581’ plants. We applied both compounds three times a week from the first visual indication of developing capitula buds until the full maturity of the capitula and florets. Unfortunately, no significant effect on the percentage of disc florets was observed for either BL or BZ treatment, even though BL treatment did increase ray floret ligule length, consistent with observations in gerbera [15] (Figure S5A–C). However, some capitula treated with BZ had a much higher disc:ray ratio than the mock-treated capitula, suggesting that the treatment had only been effective in a few cases. We, therefore, set up a second experiment with many more plants, applying fresh solutions of BZ precisely into the buds thrice a week, and compared the phenotypes of BZ-treated capitula with those of mock-treated capitula. This resulted in a clear and significantly increased percentage of disc florets (Figure 4F and Figure S5D), in accordance with the fact that lower expression of *CmDWF1* increases the disc:ray floret ratio. Besides this effect on the capitulum, there was an effect on the size of the treated plants. Two weeks after the start of the treatment, the plants were more compact and shorter. This difference in length disappeared later during development. Overall, the differential expression of BR-related genes observed in M1 and M2, the effect of downregulating *CmDWF1*, and the increased percentage of disc florets upon BZ treatments provide substantial evidence for a role of BR in the determination of the disc:ray floret ratio in chrysanthemums.

## 3. Discussion

In this study, we showed that the disc:ray floret ratio in the chrysanthemum capitulum is highly correlated to seed set efficiency. To identify genes and molecular pathways underlying this phenomenon, two chrysanthemum mutants with a higher disc:ray floret ratio were acquired. Differential transcriptomics for these varieties and their respective mutants showed that hundreds of genes were differentially expressed at early developmental stages before the appearance of visible differences between ray and disc florets in the capitulum. Downregulation by an RNAi-based silencing approach of two selected candidate genes emerging from these analyses confirmed the roles of the putative BR biosynthesis gene *CmDWF1* and the class IV homeodomain-leucine zipper gene *CmPDF2* in controlling the disc:ray floret ratio in chrysanthemums.

### 3.1. Pros and Cons of the Followed Transcriptomics Approach

The application of transcriptome analysis to identify genes potentially involved in the development of ray or disc florets in chrysanthemums is not new and has been performed previously, revealing roles for B-class MADS-box transcription factor genes and *CYC2-*like TCP transcription factor genes in differential development of ray and disc florets in chrysanthemums [18,20,21,22]. In most of these studies, morphologically different ray and disc florets were compared, resulting in a lack of insight into the initial molecular processes and associated genes that drive differential floret development or that establish patterning in the capitulum. For this reason, we isolated RNA from very early capitulum developmental stages, starting before any phenotypic distinction could be made between disc or ray primordia. The downside of this approach is that each sample contains hundreds of floret initials that are not completely synchronized in development and will develop either into ray or disc florets. However, by comparing mutants showing altered disc:ray floret ratios with their respective wild-type varieties, a correlation between floret type and gene expression was achieved, albeit quantitatively. Even though this quantitative effect hampered the identification of candidate genes, we successfully identified a set of differentially expressed genes, some of which were functionally studied by a reverse-genetics approach. In the future, the generated expression data could serve as a good starting point and reference for further detailed expression analyses using, e.g., single-cell sequencing-based technology [43].

### 3.2. Does BR Represent a Capitulum Patterning Hormone in Asteraceae?

The discovery of many genes involved in BR biosynthesis and signaling among the differentially expressed genes was intriguing because auxin and not BR is commonly connected with Asteraceae capitulum patterning and floret development [44,45]. The BR-related genes with differential expression include the BR-biosynthesis gene *DWARF1 (DWF1*) [30], the receptor kinases *BRI1-like2 (BRL2)* [46], and *HERK1* [33], and the AP2-ERF transcription factor *TINY2* [47], resembling a complete BR-signaling cascade. There were far more differentially expressed BR-related genes in the M2 than in the M1 mutant, particularly at stages 2 and 3, suggesting a more extensive role of BR in this genetic background. This is consistent with some additional phenotypic characteristics of the M2 plants, which were of shorter length and exhibited reduced pollen formation in the disc florets, both phenotypes previously connected to lower BR levels in other plant species [29]. In both mutants, the downregulation of *CmHERK1* variants was remarkably strong, with log2 fold changes between 3 and 5 in M1 (DN45464 and DN52920) and in M2 (DN49627, DN44593, DN68032). Possibly, this caused the effect on other BR-signaling genes via feedback mechanisms. It will definitely be interesting to further characterize the function of this *CmHERK1-*like gene(s) in capitulum patterning in the future. 

Our functional studies in transgenic lines with downregulation of *CmDWF1* and the outcomes of the BL treatments strongly suggest that high BR levels support ray floret development. This conclusion is supported by a very recent study in chrysanthemum, which showed that overexpression of *BRI1-EMS-SUPPRESSOR1 (CmBES1)*, a positive regulator of BR, stimulates ray floret identity, resulting in a decreased disc:ray floret ratio [48]. Detailed molecular analyses suggest that CmBES1 fulfills its function by repressing organ boundary genes in ray florets, such as *CUP-SHAPED COTYLEDON2 (CUC2)*. Interestingly, several organ boundary genes were implicated to be involved in the development of the radiate capitulum type in *Chrysanthemum lavandulifolium* [49]. Considering all this evidence, we conclude that BR is an important stimulus of ray floret identity and development in chrysanthemums. An intriguing question to answer is whether this is unique to chrysanthemums or more common in Asteraceae. A study in gerbera showed that exogenous BR treatment could increase ligule elongation in ray florets [15], but this seems to be a later effect. Another study in the monocot *Setaria viridis* also found that BR could influence floret identity [50]. Based on these observations, we hypothesize a more general role of BR in the patterning of capitula and establishment of ray floret identity in Asteraceae. 

### 3.3. A Defined Role for HD-ZIP IV Transcription Factors in the Regulation of Disc:Ray Floret Ratio

*PDF2* belongs to the class IV homeodomain-leucine zipper (HD-ZIP IV) gene family and, together with other family members, represented by either *HDG1, HDG2, HDG5,* or *HDG12*, is involved in the regulation of floral organ development in Arabidopsis [51]. PDF2 is important for petal and stamen development, while double *hdg pdf2-1* mutants showed homeotic conversions of petals and stamens into sepaloid and carpeloid structures, respectively. For another *pdf2* allele, *pdf2-2*, only effects on stamen number were found when combined with *hdg* mutant alleles [52]. The homeotic mutations are probably caused by the downregulation of the B-class *MADS-box* genes *APETALA3 (AP3)* and, to a lesser extent *PISTILLATA (PI)*, which are responsible for petal and stamen identity specification [51]. Detailed stage-specific investigation of expression showed that *AP3* expression was not affected in early stage 3 floral buds of the Arabidopsis *hd-zip IV* mutants but was spatially altered and reduced during the later development of petals and stamens, thereby affecting their differentiation and outgrowth [51]. Thus, it is possible that *CmPDF2* and possibly other *HD-ZIP IV* genes act as early floret identity specifying factors and regulate *AP3* expression in the chrysanthemum disc and ray florets, resulting in the differential outgrowth and unique characteristics of the two different floret types. In this case, CmPDF2 would rather be an early floret identity specifying factor than a factor regulating capitulum patterning. 

However, PDF2 and its close homolog ARABIDOPSIS THALIANA MERISTEM LAYER1 (ATML1) are also redundantly regulating epidermal cell differentiation in Arabidopsis [53,54,55]. The epidermal cell layer is the outermost cell layer of most tissues, usually covered with a cuticle and acting as a defensive layer against biotic and abiotic threats [56]. The downregulation of *PDF2/ATML1*-like genes in both mutants may thus also explain the strong overrepresentation of the GO term ‘cutin biosynthesis’ in both RNA-seq experiments. In addition to its function in plant defense, the epidermis also plays an important role in controlling plant growth, a function that is strongly connected to BR, as the dwarf phenotypes of BR-signaling mutants can be rescued by restoring BR signaling only in the outermost layer [57]. Furthermore, the epidermis also regulates the specification of different cell types in the SAM [58]. In plants, the spatial organization within the shoot apical meristem along an apical-basal axis is crucial for the initiation and maintenance of distinct cell types in the meristem, and this is in Arabidopsis regulated by HAIRY MERISTEM (HAM) transcription factors. These form a concentration gradient from the epidermis to the interior cell layers, which is mediated by ATML1 and PDF2 through the activation of miR171 [58]. The important roles of PDF2 and ATML1 in plant growth, patterning, and embryo development [59] may also explain why we could not obtain lines with a strong downregulation of *CmPDF2* in chrysanthemums. If the *PDF2/ATML1*-like genes have essential roles in chrysanthemums as well, further downregulation will probably impact plant viability too strongly. The relatively drastic phenotype obtained in lines with mild downregulation suggests that further downregulation may approximate a phenotype of natural chrysanthemum varieties, having only ray florets at the margin. To prove this, *CmPDF2* will have to be downregulated locally, however. 

### 3.4. Overall Conclusions and Implementation

Our data have shown that BR and the HD-ZIP IV gene *CmPDF2* play important roles in determining the disc:ray floret ratio in chrysanthemums, representing two cues not commonly associated with floret identity in Asteraceae. Since both have an important function in regulating the growth of organs in the epidermal L1 layer [51,57], they might even directly interact. For root hair differentiation, a model was proposed involving interactions between BR and the HD-ZIP protein GLABRA2 (GL2) in the L1 layer [60]. A similar interaction involving BR and CmPDF2 could be acting in the L1 layer of the chrysanthemum capitulum or young floret primordia steering floret differentiation. Directly downstream of this module, a different expression of organ boundary genes may establish ray or disc floret development in association with ABC-class MADS-box genes and preceding or complementing the action of the *CYC2* genes [13]. Interestingly, several organ boundary genes were suggested to be involved in capitula development or disc:ray floret ratio specification in Asteraceae evolution [49]. Specifically, *NO APICAL MERISTEM (NAM)/CUC-*like and *LOB30* were unique to forming radiate capitula, containing both disc and ray florets, compared to discoid capitula (only disc florets) or ligulate capitula (only ray florets). In line with this proposed mechanistic model, homologs of *CUC2, LOB DOMAIN CONTAINING PROTEIN15 (LBD15),* and *LBD19* had higher expression in M2 and *LATERAL ORGAN FUSION 2 (LOF2)* was more highly expressed in both M1 and M2 mutants (see Tables S1 and S5).

We discovered here that in the chrysanthemum, BR influences the floret ratio, suggesting that a gradient may be established in the capitulum. It is possible that, like for auxin in gerbera [44], BR is highest at the capitulum’s margin and lower at the center where disc florets develop. The type of floret is then dependent on a BR threshold. Moreover, lower levels would, in this case, result in more disc florets, and this is exactly what we see in the *CmDWF1* RNAi lines and *BZ*-treated plants. As auxin and BR regulate many targets in concert, a joint general action in Asteraceae capitula is plausible [61]. These findings could be used in further studies concerning both Asteraceae capitulum patterning and floret identity determination but also provide opportunities for improving seed sets in chrysanthemum varieties with high ornamental value. In particular, the finding that BR plays a role may be easily exploited in breeding to increase the disc:ray floret ratio for better fertility.

## 4. Materials and Methods

### 4.1. Determination of Average Seed Set Per Capitulum Type

The seed set was assessed for a wide range of crosses between different chrysanthemum varieties and was registered in a database at the Dutch breeding company ‘Dekker Chrysanten’. For the analysis, varieties were grouped based on the maternal disc:ray floret phenotype into ‘Daisy-type’ (DAI; mainly disc florets; 7486 crosses), ‘Half-Decorative-type’ (HDEC; equal number of ray and disc floret; 3086 crosses), and ‘Decorative-type’ (DEC; mainly ray florets; 17,987 crosses). Since a varying number of flower heads/capitula were used in the different crosses, data were normalized to seed per head to reduce variation. ANOVA (Tukey) in IBM SPSS Statistics 27 was used for statistical analysis.

### 4.2. Plant Growth Conditions and Phenotyping

Plants of varieties 1 and 2 (V1 and V2) and their corresponding mutants (M1 and M2) were grown in the greenhouse of Dekker Chrysanten B.V. in Hensbroek, the Netherlands. *C. morifolium* cultivar ‘1581’ plants (WT and transgenics) were grown at 20 °C in a growth chamber at Wageningen University & Research. Cuttings and rooted explants were transferred to short day (SD) conditions (10 h light/14 h dark) to induce flowering. The ratio of disc:ray florets has either been determined by counting all disc and ray florets on a capitulum or by cutting the capitulum in half and determining the number of both floret types on the cutting edge. The latter method was much more rapid but a bit less precise. 

### 4.3. Sampling and RNA-Extraction

Capitula samples for expression analyses were collected around 3 ZT in January and February of 2018. All samples were meticulously sorted into specific size classes based on diameter and representing the subsequent developmental stages. For V1 and M1: S0, 0.5–2 mm; S1, 2–3 mm; S2, 4–5.5 mm; S3, 6–8 mm; S4, 9–13 mm; and S5, 15–25 mm. For V2 and M2: S0, 0.5–2 mm; S1, 2–3 mm; S2, 3–5 mm; S3, 5–6 mm; S4, 7–10 mm; and S5, 10–25 mm. For all stages, three individual pools of buds were isolated as biological triplicates, consisting of two to five capitula. They were immediately frozen in liquid nitrogen and stored at −80 degrees Celsius. Samples were ground using a mortar and pestle, and total RNA was isolated using the innuPREP Plant RNA Kit from analytic-Jena. For expression analysis of the transgenic lines, RNA of stages 0–1 buds was extracted using a CTAB/LiCl protocol.

### 4.4. Library Preparation for RNA-Seq

RNA-seq library preparation was performed using the Illumina TruSeq^®^ Stranded mRNA sample preparation kit according to the manufacturer’s protocol. The low-sample protocol was used with a few modifications. Fragmentation was performed for three minutes at 94 °C sample; 13 cycles were used in the PCR amplification step. Library sample quantity and quality were determined by Qubit and fragment analyzer (average fragment size 311 bp). Then, 125 bp paired-end sequencing was performed on the Illumina HiSeq2500. Data de-multiplexing and adapter trimming were performed with bcl2fastq v2.20.0.422 using default settings. The average number of sequenced read pairs was 41,866,204 per sample, with the lowest number being 24,061,638 and the highest number 65,622,549.

### 4.5. RNAseq Data Analysis

A reference transcriptome used for mapping the reads was constructed using Trinity de novo assembly of all read pairs of V1, using default settings. The isoforms for each gene were reduced to one by selecting the most abundantly expressed isoform. Mapping and quantification were performed using RSEM and Bowtie2. Mapping of samples for V1 and M1 was performed using default settings, and for V2 and M2, bowtie2 sensitivity settings were adjusted to ‘very_sensitive’ to adjust for a higher variance compared to the reference sequence used. Differential expression analysis was performed in R using DESeq2 with normalized data, selecting the nbinomWaldTest and only focusing on genes with a maximum 0.01 Padj value, while not taking any minimum fold change into account. Sequences were compared to Arabidopsis database Araport11_genes.201606.pep by BLAST, using blastx with various e-value settings to identify homologs. The statistical overrepresentation test of biological GO-terms was performed on pantherdb.org using the Arabidopsis identifiers. The raw data of both RNA-seq experiments have been deposited at the Gene Expression Omnibus (GEO, https://www.ncbi.nlm.nih.gov/geo/) accessed on 25 March 2023 with accession numbers GSE227132 (V1/M1) and GSE227133 (V2/M2).

### 4.6. qPCR Analysis

cDNA synthesis was performed using Invitrogen superscript^®^ IV reverse transcriptase and random hexamer primers according to the manufacturer’s instructions. Real-time PCR was performed using PowerTMSYBR^®^ green master mix. *CmSAND* and *CmPGK* [62] were both used as reference genes, of which the latter was used for calculations and graphs. For disc and ray floret tissues, *CmEF-1a* was used as a reference gene [62]. PCR and measurements were done using the CFX maestro Bio-rad real-time PCR machine with 384-well plates. The significance of potential expression differences was calculated using the student *t*-test. Gene sequences and primers: *AGAMOUS (CAG1)* [63], *CmLFY* [64], and *CYC2c* [13]. The primer sequences are listed in Table S6.

### 4.7. Cloning of Constructs for Transformation

RNAi constructs were generated using a Goldengate approach [65], with vectors from the Golden Gate MoClo Plant Parts set [66]. Different modules were coupled in the binary level 2 vector pICSL4723. A fragment consisting of 300–400 bp of the coding sequence of the respective chrysanthemum genes was selected in a region of low homology with other sequences. Additionally, an intron containing 417 bp of the intron from vector pK7GWIWG2 [67] was cloned, excluding Bpi/BsaI sites. The three fragments were PCR-amplified from the plasmid template (intron) and from chrysanthemum cDNA (gene fragments) and connected in a ‘fragment-intron-inverted fragment-module’ into pICH47761, using the GoldenGate approach. Additionally, the following modules were combined in the level 2 vector: CaMV35S:NPTII in pICH47732, CaMV35S:GFP in pICH47742 (to assess transformation success), PcUbi in pICH47751, RNAi fragments in pICH47761, terminator tChrRbcS in pICH47772, and End-linker pELE5 [66]. Level 1 constructs were prepared using BsaI, and level 2 constructs using BpiI. The primers used for generating the modules that were newly prepared for this study can be found in Table S6. After level 2 assembly, the resulting constructs were checked using restriction digestion and sequencing and transformed into *Agrobacterium tumefaciens* strain AGL0.

### 4.8. Plant Transformation

*Chrysanthemum morifolium* cultivar ‘1581′ was used for transformation and regularly propagated in vitro to provide young leaves for transformation. *Agrobacterium tumefaciens* AGL0 was inoculated in 15 mL LB with kanamycin, gentamicin, rifampicin, and 100 µM acetosyringone and grown overnight in a shaker at 28 °C. The *Agrobacterium* culture was then centrifuged for 20 min at 3500 rpm and resuspended in inoculation medium (1/1 MS, 3% sucrose, 1 mg/L 6-benzylaminopurine (BAP), 1 mg/L indole-3-acetic acid (IAA) and 100 µM acetosyringone) to an OD600 of 0.5. Young leaves were cut while submerged in inoculation medium to ~0.3 cm^2^ squares (including larger leaf nerves) and incubated for 15 min. Leaf explants were then dried on filter paper and placed bottom-up on co-cultivation medium (3% sucrose, 1 mg/L 1-naphthaleneacetic acid (NAA), 7 g/L plant agar, and 100 µM acetosyringone). The explants were incubated in a climate chamber (16 h light, 21 °C) under two layers of filter paper for four days, after which they were transferred to a selection medium (1/1 MS, 3% sucrose, 1 mg/L BAP, 1 mg/L NAA, 25 mg/L kanamycin, 500 mg/L Carbenicillin, 7 g/L plant agar). Explants were transferred to a fresh selection medium every three weeks. Transformed shoots were recognized by expression of GFP from the 35S:GFP module. After 6–8 weeks, the first transformed shoots were harvested and transferred to propagation medium (0.5 MS basal salt mixture and full MS vitamins, 3% sucrose, 8 g/L lab M agar, and 0.1 mg IAA (pH 5.8)). In each transformation, a control plate was added with non-transformed explants to produce control WT plants for expression analyses and phenotyping. Transformed plants were grown until they developed roots and thereafter moved to soil in a growth chamber at 20 °C. Plants were kept underneath a plastic sheet for one week in long-day conditions (LD; 16 h light) and then moved to short-day conditions (SD; 10 h light). For *CmPDF2* and *CmDWF1*, clones were produced from the primary transformants through cuttings.

### 4.9. Brassinolide and Brassinazole Treatments

5 mM stock concentrations of epibrassinolide (BL) and brassinazole (BZ) (Sigma) were prepared in 96% ethanol and stored at −80 °C in aliquots. These were diluted in milliQ to a concentration of 10 µM (BL) and 50 µM (BZ) and used fresh every treatment. Silwet (0.2%) was added to the working solutions. The mock solution was prepared similarly, but by adding 96% ethanol without compound in the same dilution. Then, 100 µL of the solution was applied by pipetting on individual apical capitulum meristems. This treatment was repeated three times a week. Treatment started one week after the transfer of the plants to inductive short-day conditions and ended when the florets were fully opened.

## Data Availability

The raw data of the RNA-seq experiments is available via the GEO data repository (https://www.ncbi.nlm.nih.gov/geo/) accessed on 25 March 2023 under accession numbers GSE227132 (V1/M1) and GSE227133 (V2/M2).

## Supplementary Materials

The following supporting information can be downloaded at: https://www.mdpi.com/article/10.3390/ijms24087655/s1.
