# Supplementary figures and images for: HD-ZIP Transcription Factors and Brassinosteroid Signaling Play a Role in Capitulum Patterning in Chrysanthemum
Source: Int J Mol Sci. 2023 Apr 21;24(8):7655. doi: 10.3390/ijms24087655 (PMC10141471; doi:10.3390/ijms24087655)

Figure S1

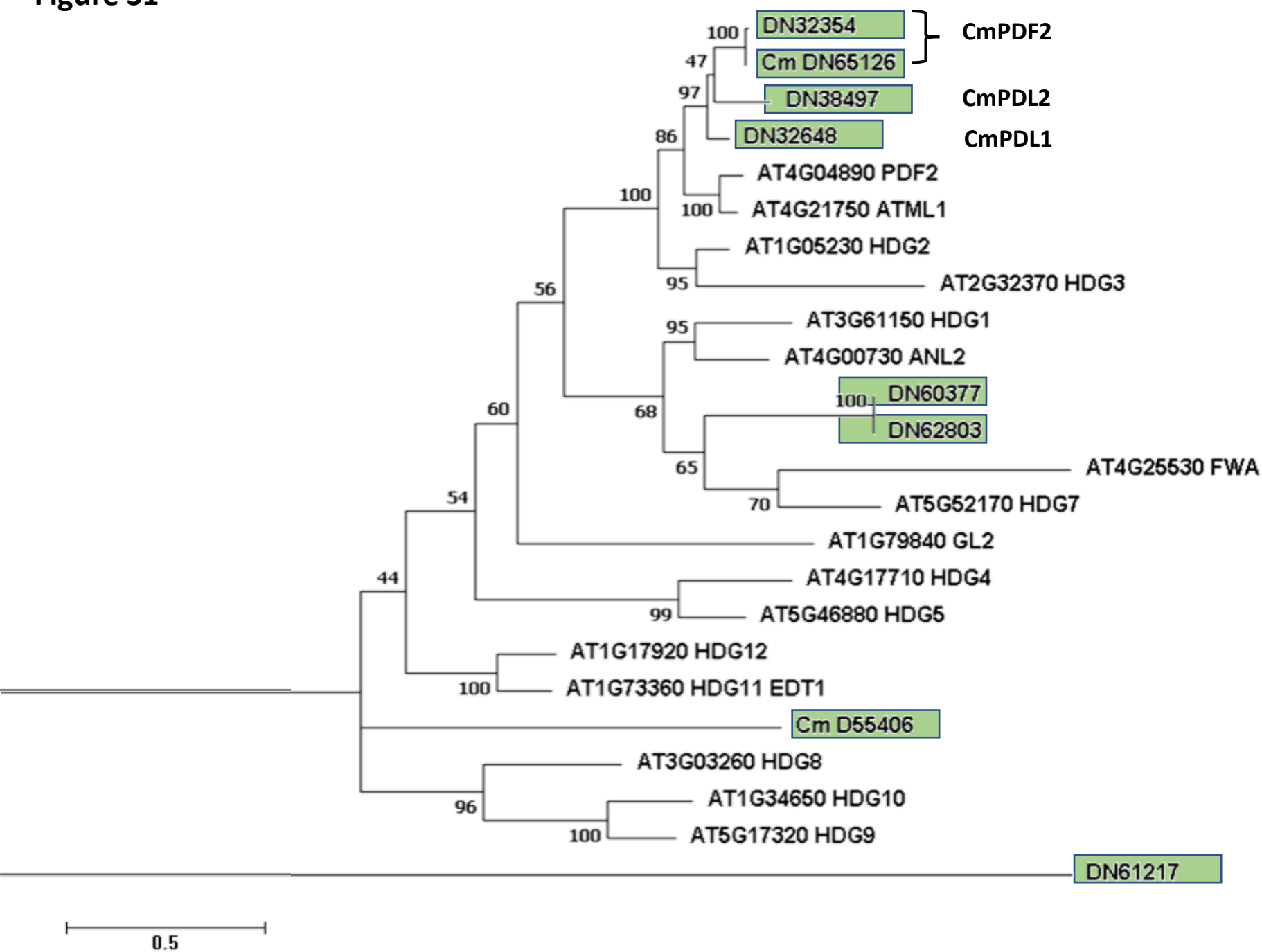

Figure S2

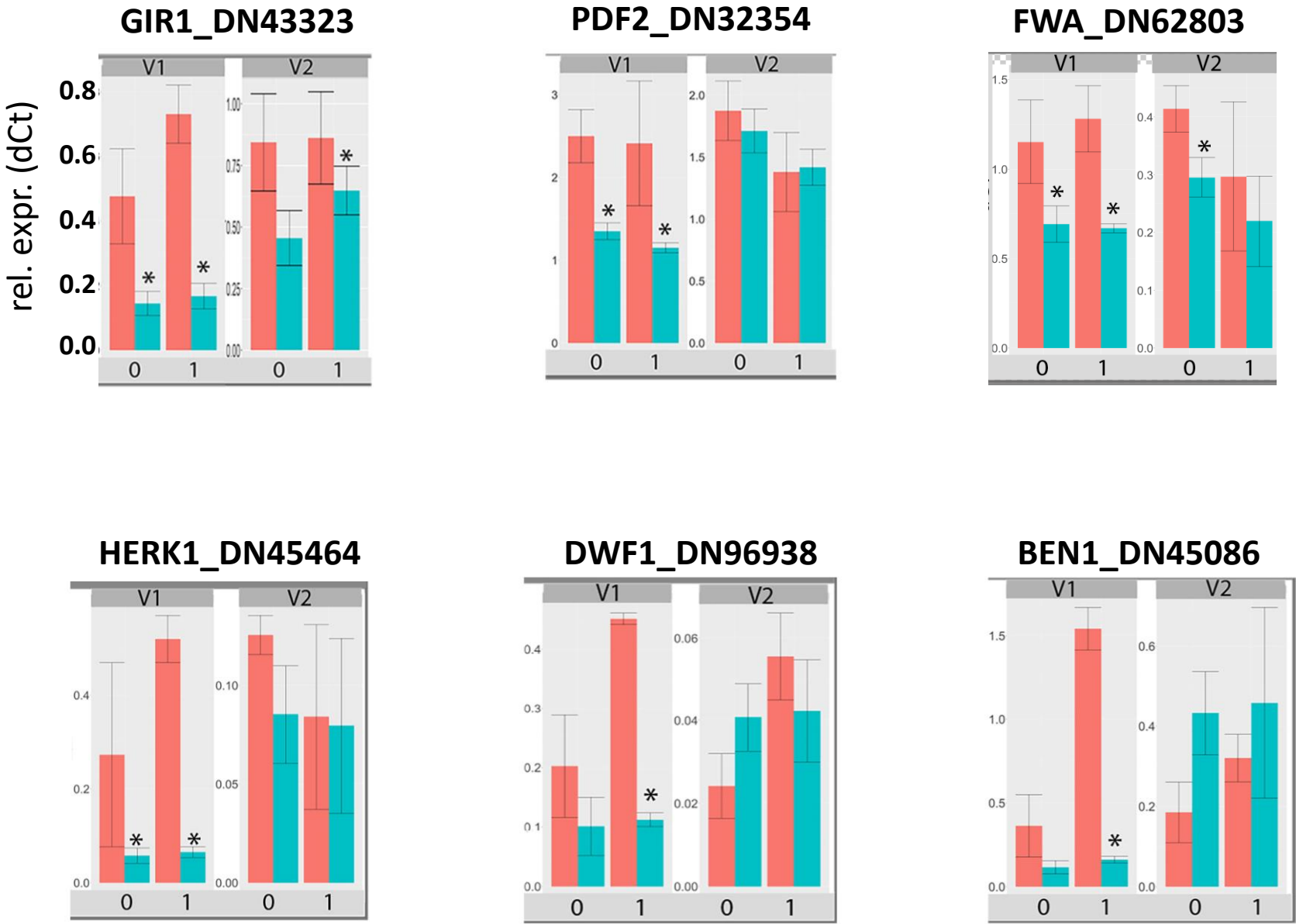

Figure S3

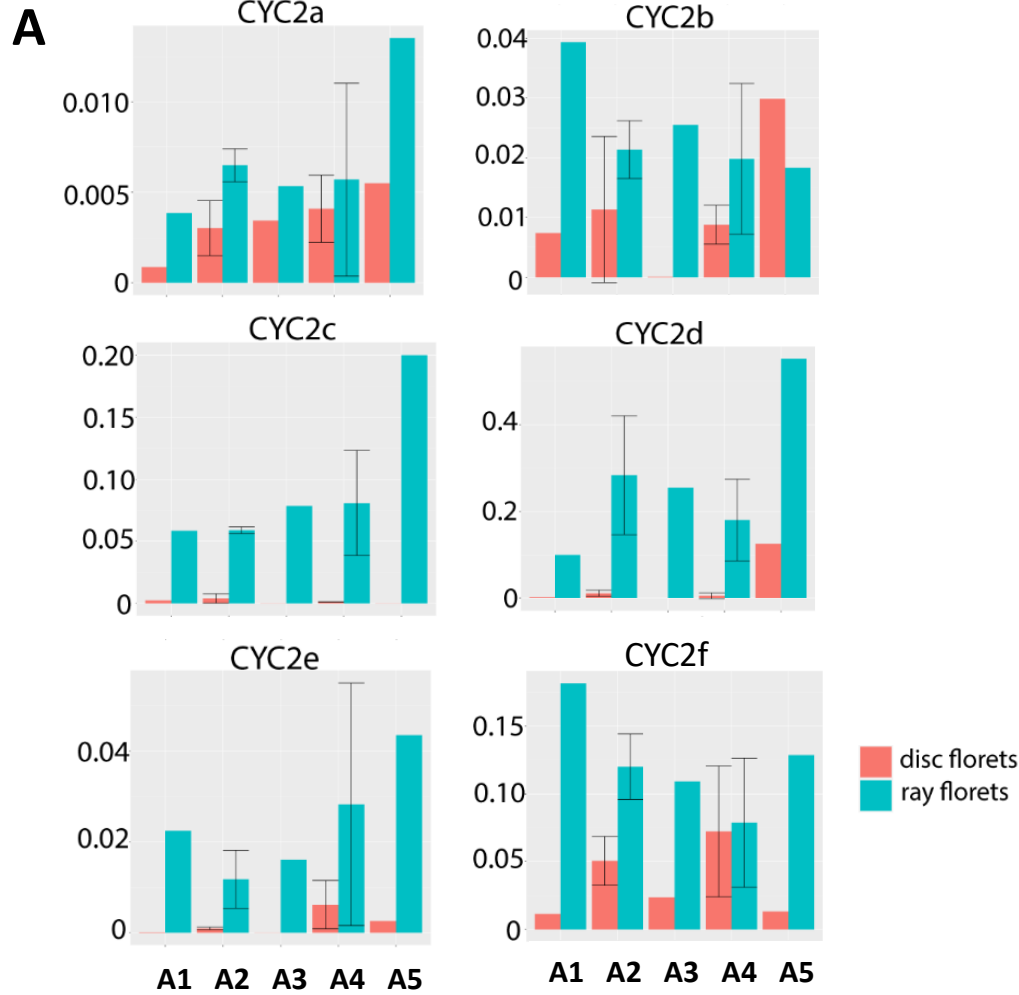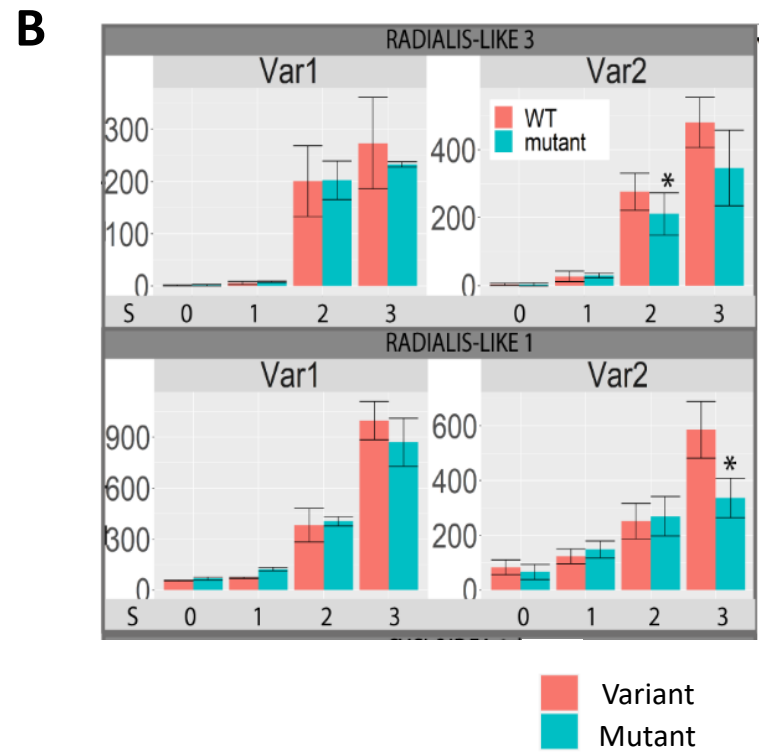

Figure S4

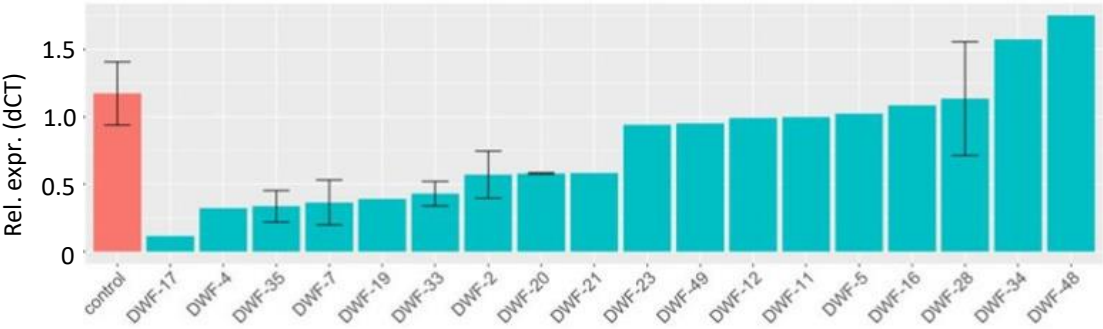

CmDWF1  
RNAi lines

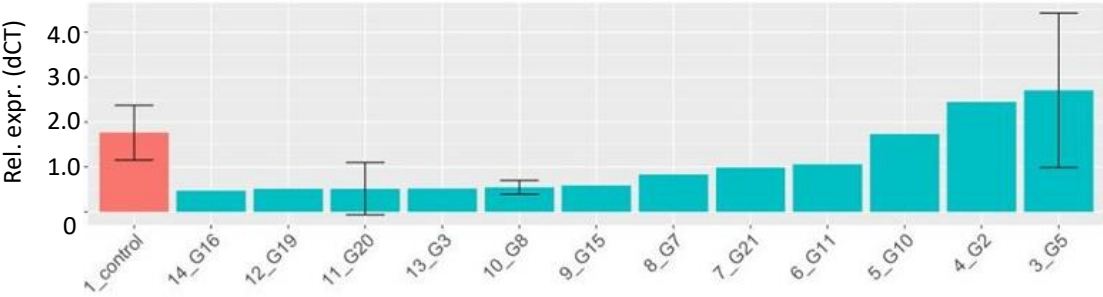

CmGIR1  
RNAi lines

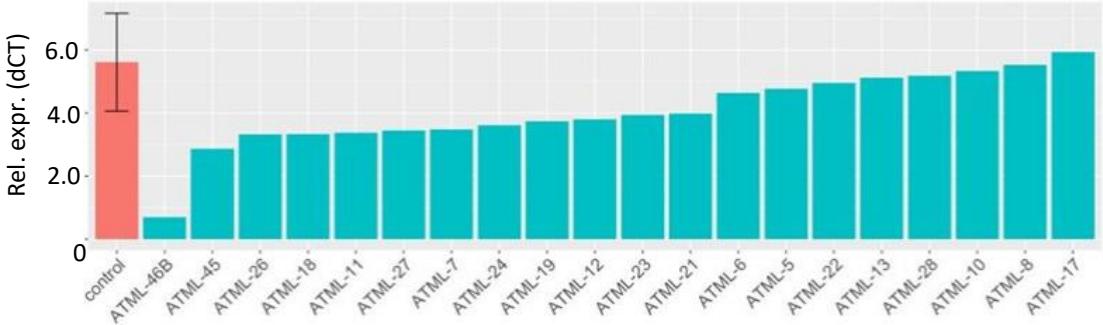

CmPDF2  
RNAi lines

Figure S5

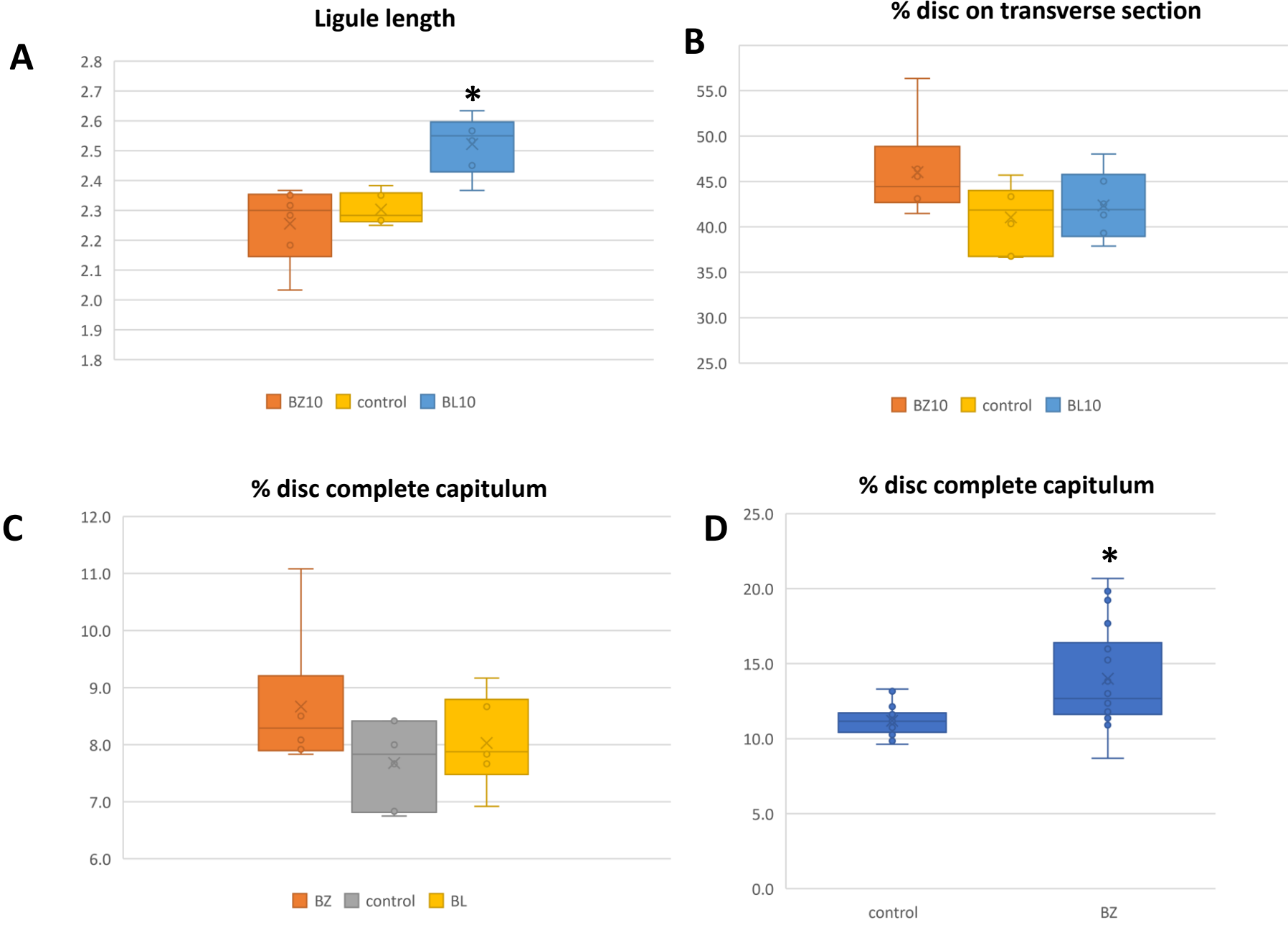

Supplement: Supplementary file 1 [file ijms-24-07655-s001.zip › Supplementary Figures.pdf]
